# A Machine Learning–Based Model to Predict Overactive Bladder Risk Among US Women: Evidence From the National Health and Nutrition Examination Survey 2011-2018

**DOI:** 10.2196/80133

**Published:** 2026-08-12

**Authors:** Guoqiang Huang, Shuangquan Lin

**Affiliations:** 1 Department of Urology, The Second Affiliated Hospital of Nanchang University, 1 Mingde Road, Nanchang, Jiangxi Province, 330200, China, 86 15727561896

**Keywords:** overactive bladder, women, machine learning, predictive modeling, National Health and Nutrition Examination Survey, NHANES, cross-sectional study

## Abstract

**Background:**

Overactive bladder (OAB) is a prevalent condition, particularly among women, characterized by urinary urgency, often accompanied by frequency and nocturia. Traditional risk prediction methods for OAB are limited, as they fail to fully integrate multidimensional risk factors, including female reproductive history. Machine learning offers potential for enhanced predictive accuracy by using large-scale datasets like the National Health and Nutrition Examination Survey (NHANES).

**Objective:**

This study aimed to develop and validate a machine learning–based model to predict OAB risk in women, incorporating reproductive and sociodemographic factors, and to identify key predictors using interpretable methods.

**Methods:**

This retrospective observational study analyzed data from 7884 participants across 4 consecutive cycles (2011‐2018) of the National Health and Nutrition Examination Survey. LASSO (least absolute shrinkage and selection operator) regression and univariate and multivariate logistic regression analyses were applied to identify key variables in the training set. Fourteen variables were selected via LASSO regression for model construction, among which 5 continuous predictors—age, BMI, ratio of family income to poverty threshold (PIR), age at menarche, and number of vaginal deliveries—remained significantly associated with OAB in multivariable logistic regression and were used in the subsequent dose-response analysis. The SHAP (Shapley Additive Explanations) method interpreted the optimal model, and restricted cubic spline (RCS) curves were used for dose-response analysis.

**Results:**

Five continuous predictors—age, BMI, PIR, age at menarche, and number of vaginal deliveries—remained significantly associated with OAB in multivariable logistic regression and were carried forward to the dose-response analysis. Among the 11 ML models, random forest (RF) demonstrated the highest predictive performance. The random forest model achieved an AUROC (area under the receiver operating characteristic curve) of 0.8536 (95% CI 0.8435‐0.8638) in the training set and 0.6999 (95% CI 0.6768‐0.7212) in the test set, indicating moderate predictive capability. SHAP analysis identified age, BMI, and normal menstrual status as the top 3 contributors to OAB risk. Both RCS and SHAP analyses revealed a positive association of age and BMI with OAB risk and a negative association with PIR. Additionally, RCS showed that the risk of OAB was higher with an earlier age at menarche and a greater number of vaginal deliveries.

**Conclusions:**

Integrating ML with SHAP interpretability provides a predictive tool for OAB with moderate predictive accuracy; further external validation and clinical utility assessment are warranted before clinical application.

## Introduction

Overactive bladder (OAB) is characterized by urinary urgency with or without urinary incontinence in the absence of urinary tract infection or other pathology [1]. It is frequently accompanied by increased daytime urinary frequency and nocturia. OAB is estimated to affect millions of people worldwide and is more prevalent in women than in men [2,3]. Despite the high prevalence of OAB, accurate prediction of OAB risk remains a challenge. Traditional methods rely heavily on clinical symptoms and a basic physical examination, often failing to fully consider all influencing factors. Understanding the risk factors associated with OAB, especially in the context of women's health, is crucial for developing effective prevention and management strategies.

Previously, the majority of research on OAB mainly focused on other related factors, such as BMI [4], diabetes [5,6], and inflammatory indicators [7,8]. However, research on female reproductive factors remains insufficient and represents a significant research gap. Previous studies have shown that gender-specific differences exist in the structure and function of the innervation of the female urethra and bladder, which may lead to bladder hypersensitivity and detrusor overactivity [9]. In terms of hormonal levels, fluctuations in estrogen are closely associated with the onset and severity of OAB symptoms [10]. In particular, women in perimenopause experience a sharp change in estrogen levels, significantly increasing their risk of developing OAB [11]. Furthermore, during pregnancy, significant changes occur in the structure and function of the pelvic floor, which is likely to be a key trigger for the onset of OAB [12].

To address this gap, this study focuses specifically on underinvestigated female reproductive factors (such as age at menarche, number of pregnancies, menopausal status, etc) using machine learning (ML) approaches. The ability of ML to analyze large-scale, complex data; extract underlying patterns; and construct accurate predictive models significantly improves the efficiency and accuracy of disease prediction compared to traditional statistical methods. Compared with previous studies that had limitations such as a small number of participants and regional or local biases, our research used the National Health and Nutrition Examination Survey (NHANES) database, which is based on national population survey data in the United States. The NHANES database covers a wide range of populations and has excellent representativeness, enabling it to comprehensively reflect the health conditions and reproductive characteristics of women of different ages, races, and regions, thus providing a solid data foundation for accurately constructing a risk prediction model for female OAB. By integrating multidimensional reproductive-related variables, this study aims to establish a comprehensive and accurate risk prediction model for OAB, with the goal of identifying high-risk female populations at an early stage and providing a basis for formulating personalized prevention and intervention strategies.

## Methods

### Ethical Considerations

All procedures of the National Health and Nutrition Examination Survey (NHANES) involving human participants, materials, or data were conducted in accordance with the Declaration of Helsinki and approved by the National Center for Health Statistics (NCHS) Ethics Review Board (Protocol #2011-17). Written informed consent was obtained from all participants. The study did not require clinical trial registration, and the survey data are publicly accessible for researchers and data users worldwide [13].

### Data Source and Study Population

The Transparent Reporting of a multivariable prediction model for Individual Prognosis or Diagnosis with Artificial Intelligence (TRIPOD+AI) guidelines (Checklist 1) were adhered to in the conduct of this study [14]. The NHANES, a nationwide survey using a stratified, multistage sampling process, provided the data for this investigation, assessing the nutritional and medical conditions of individuals across all age groups in the United States. The NHANES, established by the NCHS, is a comprehensive national survey designed to evaluate the nutritional status and overall health of the US population. The study’s design and data collection methods were reviewed and approved by the NCHS Ethics Review Board. Comprehensive information regarding ethical approval can be found on the Centers for Disease Control and Prevention website. Data from 4 consecutive survey cycles (2011‐2018) were analyzed for OAB.

### OAB Assessment

Urgency urinary incontinence and nocturia are the principal symptoms of OAB, which is a prevalent clinical condition in the field of urology. OAB symptoms in the population were systematically evaluated using the renal disease-urology questionnaire from NHANES, and the Overactive Bladder Symptom Score was used to quantify the severity of OAB symptoms. In this study, OAB was defined on the basis of self-reported symptoms, with a total score of 3 or higher on the Overactive Bladder Symptom Score indicating OAB; because NHANES does not include urinalysis or urine culture data, urinary tract infection and other pathologies that can cause similar symptoms could not be excluded. Urgency urinary incontinence and nocturia are among the multiple symptoms taken into account by the Overactive Bladder Symptom Score [15], a reliable assessment tool, and are translated into a specific score. According to the scoring results, patients are regarded as having OAB when their total score reaches 3 points or higher (Figure S5 in Multimedia Appendix 1).

### Covariate Assessment

Aiming to explore the risk factors associated with female OAB, female reproductive history—an integral part of women’s lives—was integrated as a research variable in this study. Variables were obtained with the assistance of the reproductive health questionnaire. The reproductive health questionnaire comprised menstrual history, pregnancy history, use of birth control pills, and other relevant reproductive aspects. At the mobile examination center, the questionnaire was administered by trained interviewers through the Computer Assisted Personal Interview system. Among these variables, age at menarche was the response furnished by the participants to the query: “At what age did the first menstrual period occur?” Normal menstrual status was the response to the question, “Did you have regular periods in the past 12 months?” (Yes/No). A positive response (Yes) denoted the occurrence of normal menstruation during the past 12 months, whereas a negative response (No) indicated its absence. The number of vaginal deliveries was the response to the question: “How many vaginal deliveries have you had?” Contraceptive use was the response to the question, “Have you ever taken birth control pills?” (Yes/No). Pregnancy diabetes diagnosis was the response to the question, “During pregnancy, were you informed that you had diabetes?” (Yes/No).

Furthermore, drawing on databases and clinical expertise, a host of additional variables were incorporated in relation to diverse modules within NHANES (Table S3 in Multimedia Appendix 1), including demographic details (age, race, ratio of family income to poverty threshold [PIR], and education), , questionnaire-derived data (smoking, alcohol, and physical activity), physical examination metrics (BMI, systolic blood pressure [SBP], and diastolic blood pressure), and laboratory examination outcomes (white blood cell count, lymphocyte count, monocyte count, platelet count, neutrophil count, and glycohemoglobin). Neutrophil count was collected and processed, and was entered into the LASSO regression as one of the 23 candidate variables; its coefficient was shrunk to zero at the selected λ (0.00550), and it was not retained among the 14 variables selected for model construction.

### Data Preprocessing

Variables with more than 30% missing data were excluded. Subsequently, k-nearest neighbor (KNN) imputation was performed using the R package DMwR2. The parameters were set as follows: *K*=15, with the mode for categorical variables specified as meth=“most” and the mean used for continuous variables. Furthermore, a comparison between the data before and after imputation was carried out (Multimedia Appendix 1), and the results demonstrated that no statistically significant difference was caused by the imputation (*P*>.05). The missing data rates for all analyzed variables were relatively low, uniformly remaining below 10% (ranging from 0% to 9.5%), ensuring the validity of subsequent KNN imputation. In addition, the correlation analysis of the continuous variables was performed using correlation heat maps (Figure S2 in Multimedia Appendix 1) to inspect multicollinearity. All 23 candidate variables were subsequently entered into the LASSO regression, and collinearity among the 14 variables retained by LASSO was checked in the data preprocessing for the machine learning models. All categorical variables were factorized before modeling, and for multiclass variables, dummy variables were set up. To maintain the continuity of the data, no classification was imposed on the continuous variables.

### Selection of Variables

Given that high-dimensional data tend to complicate ML algorithm performance, before model construction, LASSO (least absolute shrinkage and selection operator) regression [16], a method that involves selecting variables and reducing model complexity through parameter tuning to mitigate the risk of overfitting, was used on the training set. This approach uses the parameter λ to regulate model complexity, ultimately resulting in a model with fewer variables. The LASSO regression was implemented to identify the most critical factors for model inclusion. To determine the optimal λ, 10-fold cross-validation was performed, with the minimum λ value used for variable selection. The Shapley Additive Explanations (SHAP) values of the 5 continuous predictors that remained significantly associated with OAB in multivariable logistic regression (age, BMI, PIR, age at menarche, and number of vaginal deliveries) were investigated to ascertain the dose-response association between independent risk variables and OAB. These values were evaluated using a restricted cubic spline (RCS) function. Additionally, univariable and multivariable logistic regression (LR) models were used to explore the association between the variables and OAB, with odds ratios and 95% CIs calculated in the training set. Finally, variables that were non-zero coefficients in the LASSO regression were selected for constructing the ML model.

### Model Development

To predict the risk of OAB in women, we trained 11 ML models: LR, ridge regression, support vector machine, elastic net, eXtreme gradient boosting (XGBoost), random forest (RF), light gradient boosting machine, KNN, decision tree, multilayer perceptron, and naive Bayes. To ensure optimal performance and reliability, we tuned hyperparameters using Bayesian optimization combined with 5-fold cross-validation, selecting the best model configuration based on generalization performance across different hyperparameter settings. Model performance was comprehensively evaluated using more than 10 metrics, including area under the receiver operating characteristic curve (AUROC), precision, *F*_1_-score, recall, accuracy, area under the precision-recall (PR) curve, and decision curve analysis. Finally, the model that showed the best and most robust performance on the test set was selected as the final prediction model, and its interpretability was analyzed using SHAP values.

### Model Interpretability

It is difficult to explain ML models. The “black box” problem is solved by the SHAP method, a game theory–based approach that prioritizes the significance of input features and provides an explanation for the output of the prediction model. The SHAP technique increases the model’s interpretability and transparency by determining each feature’s contribution to the prediction outcomes while offering both local and global explanations [17]. The SHAP technique, which gives each variable a corresponding attribution value (SHAP value), was used to clarify the final model with the highest effectiveness. These SHAP values quantify each feature’s contribution to the model’s prediction. A SHAP summary plot was created to show each feature’s contribution to the model. Furthermore, SHAP interaction analysis was performed to explore the pairwise interactions among the main variables, thereby revealing how the effect of one variable on the prediction depends on the value of another.

### Statistical Analysis

In the baseline analyses, the Shapiro-Wilk test was used to assess the normality of continuous variables. To compare continuous variables that exhibited a normal distribution, the Student *t* test was used. These variables were presented in the form of mean (SD). For the comparison of continuous data featuring skewed distributions, either the Kruskal-Wallis *H* test or the Mann-Whitney *U* test was used. Such data were presented as medians along with IQRs. The chi-square test was applied to compare categorical variables. These variables were presented as numbers in conjunction with percentages. Additionally, dose-response relationships for all continuous variables in the ML models were analyzed using RCS curves. All statistical analyses were executed using R version 4.4.2. The development of all ML models was facilitated by the R package “tidymodels.” A 2-tailed *P*-value less than .05 was considered statistically significant.

## Results

### Patient Characteristics

**Figure 1.**
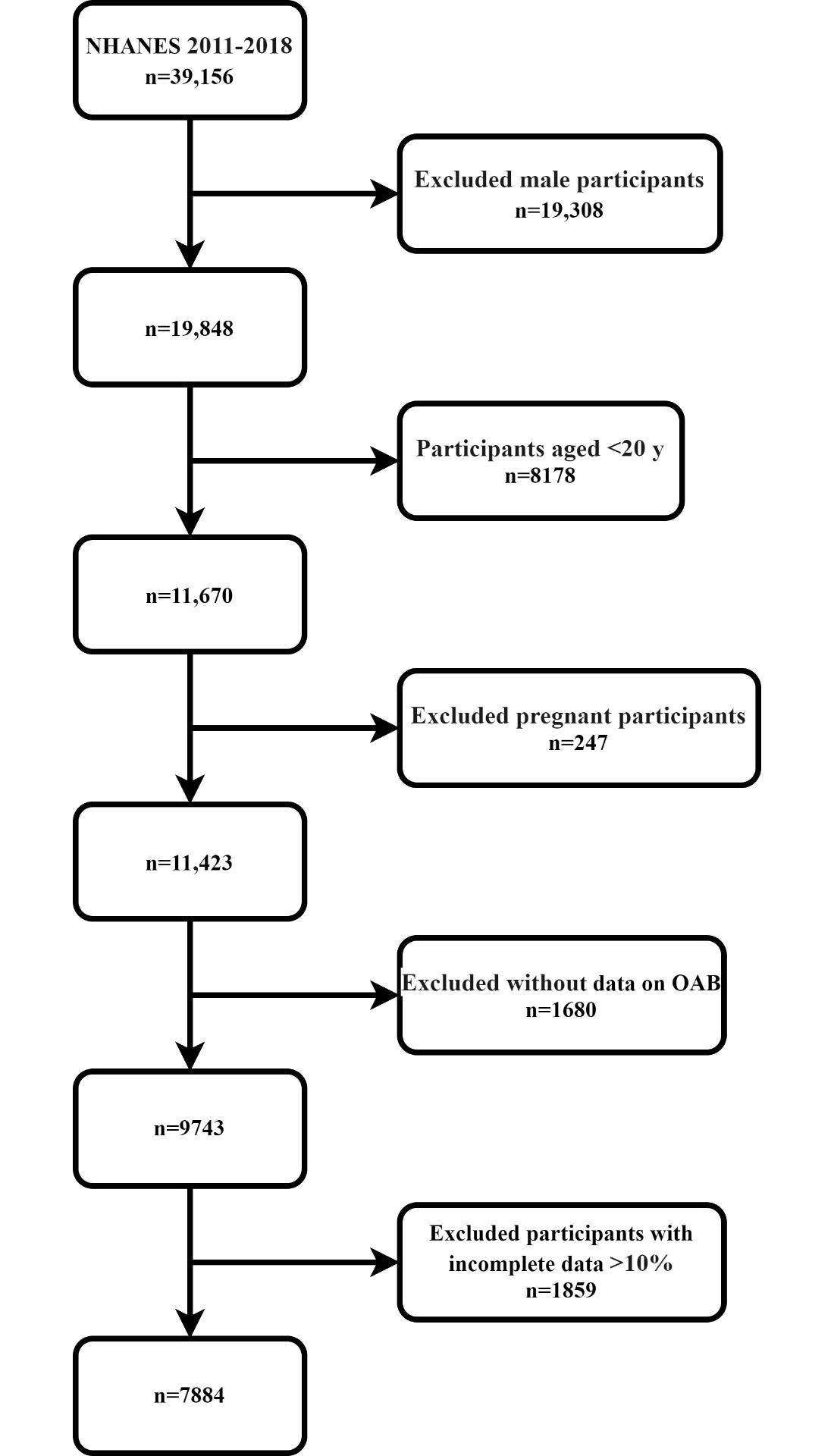
Flowchart of the study design. OAB: overactive bladder; NHANES: National Health and Nutrition Examination Survey.

A total of 7884 participants were enrolled in the study, from a total of 39,156 individuals, comprising a training set (70%, n=5519) and a test set (30%, n=2365; Figure 1). As illustrated in Table 1, significant differences were identified in most variables between the OAB and non-OAB groups within the training set (*P*<.05). For example, it was found that patients with OAB exhibited an advanced age (61 y vs 47 y in the training set and 61 y vs 48 y in the test set) and possessed a relatively higher BMI (30.7 kg/m^2^ compared to 28.0 kg/m^2^ in the training set and 31.1 kg/m^2^ compared to 28.2 kg/m^2^ in the test set). Notably, the OAB group exhibited higher blood pressure values and a larger proportion of individuals with “normal menstruation” marked as “No,” which may reflect the older age of patients with OAB.

**Table 1.** Baseline characteristics of the training and test sets.

Characteristics and variables	Training set (N=5519)			Test set (N=2365)		
	No (n=3781)	Yes (n=1738)	Overall *P* value	No (n=1602)	Yes (n=763)	Overall *P* value
Age (y), median (IQR)	47.0 (35.0-61.0)	61.0 (48.0-71.0)	<.001	48.0 (36.0-62.0)	61.0 (47.0-71.5)	<.001
Race, n (%)			<.001			.001
Mexican American	529 (14.0)	233 (13.4)		239 (14.9)	104 (13.6)	
Other Hispanic	461 (12.2)	182 (10.5)		182 (11.4)	75 (9.83)	
Non-Hispanic White	1374 (36.3)	734 (42.2)		585 (36.5)	317 (41.5)	
Non-Hispanic Black	802 (21.2)	431 (24.8)		360 (22.5)	196 (25.7)	
Other race	615 (16.3)	158 (9.09)		236 (14.7)	71 (9.31)	
Education, n (%)			<.001			.002
Below high school	762 (20.2)	416 (23.9)		323 (20.2)	189 (24.8)	
High school or comparable	778 (20.6)	404 (23.2)		345 (21.5)	187 (24.5)	
College or above	2241 (59.3)	918 (52.8)		934 (58.3)	387 (50.7)	
Marital status, n (%)			<.001			<.001
Married or living with partner	2197 (58.1)	858 (49.4)		940 (58.7)	359 (47.1)	
Widowed, divorced, or separated	1584 (41.9)	880 (50.6)		662 (41.3)	404 (52.9)	
PIR^a^, median (IQR)	2.20 (1.17-3.92)	1.98 (1.08-3.40)	<.001	2.02 (1.06-3.74)	1.88 (1.06-3.13)	.02
Smoking, n (%)			<.001			.002
Never	2541 (67.2)	1062 (61.1)		1053 (65.7)	450 (59.0)	
Former	624 (16.5)	397 (22.8)		289 (18.0)	180 (23.6)	
Current	616 (16.3)	279 (16.1)		260 (16.2)	133 (17.4)	
Alcohol, n (%)			.23			.13
No	3548 (93.8)	1646 (94.7)		1506 (94.0)	704 (92.3)	
Yes	233 (6.16)	92 (5.29)		96 (5.99)	59 (7.73)	
Physical activity, n (%)			.39			.85
Inactive	2402 (63.5)	1138 (65.5)		1042 (65.0)	508 (66.6)	
Moderate	893 (23.6)	389 (22.4)		330 (20.6)	153 (20.1)	
Vigorous	90 (2.38)	46 (2.65)		45 (2.81)	18 (2.36)	
Both moderate and vigorous	396 (10.5)	165 (9.49)		185 (11.5)	84 (11.0)	
Age at menarche (y), median (IQR)	13.0 (12.0-14.0)	13.0 (12.0-14.0)	.06	13.0 (12.0-14.0)	13.0 (12.0-14.0)	.22
Normal menstruation, n (%)			<.001			<.001
No	1890 (50.0)	1330 (76.5)		833 (52.0)	554 (72.6)	
Yes	1891 (50.0)	408 (23.5)		769 (48.0)	209 (27.4)	
No of vaginal deliveries, median (IQR)	2.00 (1.00-3.00)	2.00 (1.00-4.00)	<.001	2.00 (1.00-3.00)	2.00 (1.00-3.18)	<.001
Contraceptive, n (%)			.77			.65
No	1182 (31.3)	551 (31.7)		529 (33.0)	244 (32.0)	
Yes	2599 (68.7)	1187 (68.3)		1073 (67.0)	519 (68.0)	
Pregnancy diabetes, n (%)			.54			.96
No	3449 (91.2)	1590 (91.5)		1467 (91.6)	702 (92.0)	
Borderline	26 (0.69)	16 (0.92)		10 (0.62)	4 (0.52)	
Yes	306 (8.09)	132 (7.59)		125 (7.80)	57 (7.47)	
Diabetes, n (%)			<.001			<.001
No	3296 (87.2)	1298 (74.7)		1372 (85.6)	585 (76.7)	
Borderline	95 (2.51)	72 (4.14)		41 (2.56)	24 (3.15)	
Yes	390 (10.3)	368 (21.2)		189 (11.8)	154 (20.2)	
Glycohemoglobin (%), median (IQR)	5.50 (5.20-5.80)	5.70 (5.40-6.20)	<.001	5.50 (5.20-5.90)	5.70 (5.40-6.10)	<.001
WBC^b^ (×10^9^/L), median (IQR)	6.90 (5.70-8.40)	6.90 (5.70-8.40)	.36	6.90 (5.70-8.40)	6.90 (5.60-8.45)	.69
Lymphocytes (×10^9^/L), median (IQR)	2.10 (1.70-2.60)	2.10 (1.70-2.60)	.37	2.10 (1.70-2.70)	2.00 (1.60-2.60)	<.001
Monocytes (×10^9^/L), median (IQR)	0.50 (0.40-0.60)	0.50 (0.40-0.60)	<.001	0.50 (0.40-0.60)	0.50 (0.40-0.60)	.23
Platelets (×10^9^/L), median (IQR)	246 (208-291)	239 (205-286)	.006	249 (214-291)	238 (205-277)	<.001
BMI (kg/m^2^), median (IQR)	28.0 (23.7-33.4)	30.7 (26.1-36.1)	<.001	28.2 (24.0-33.3)	31.1 (26.2-36.5)	<.001
SBP^c^, median (IQR)	118 (109-131)	127 (115-140)	<.001	120 (109-133)	126 (116-139)	<.001
DBP^d^, median (IQR)	70.0 (63.0-76.5)	69.0 (62.0-77.0)	.06	70.0 (64.0-77.0)	69.0 (62.0-76.0)	.02

a PIR: ratio of family income to poverty threshold.

b WBC: white blood cell.

c SBP: systolic blood pressure.

d DBP: diastolic blood pressure.

### Model Variable Selection

**Figure 2.**
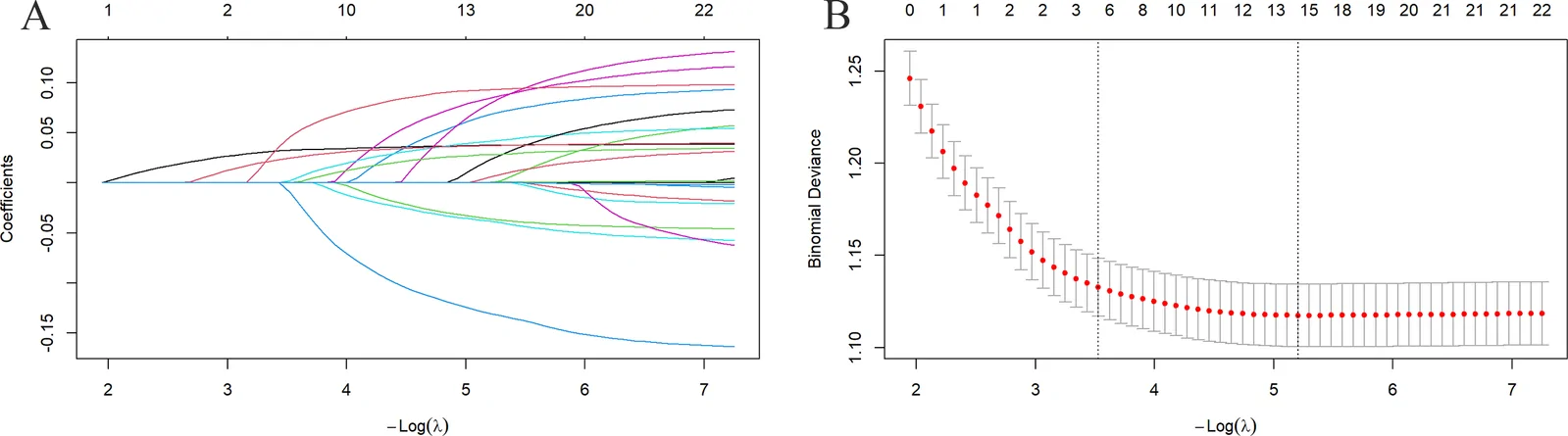
Results of the LASSO (least absolute shrinkage and selection operator) method for variable selection. (A) LASSO coefficient curves for 23 candidate variables. Coefficients are plotted on the y-axis, and −log(λ) is shown on the x-axis. As log(λ) increases, the tuning parameter strengthens, shrinking the absolute magnitude of coefficients toward zero, which leads to their elimination. (B) Ten-fold cross-validation with the minimum criterion selected the optimal λ value. The relationship between −log(λ) and binomial deviance is shown. The dashed vertical lines indicate the minimum binomial deviance (right line; λ=0.00550, corresponding to −log(λ)≈5.2) and 1 SE from the minimum (left line; λ=0.02933, corresponding to −log(λ)≈3.5). Variable selection was based on the minimum criterion: 14 predictor variables corresponding to the λ value with the minimum binomial deviance (λ=0.00550) were retained*.*

As shown in Figures 2A and 2B, LASSO regression was performed to select robust risk factors. Fourteen variables—age, marital status, PIR, smoke, physical activity, age at menarche, normal menstrual status, number of vaginal deliveries, contraceptive use, pregnancy diabetes, diabetes, glycohemoglobin, BMI, and SBP—demonstrated significant statistical associations and retained non-zero coefficients in the LASSO model. These 14 variables were, therefore, all included in the construction of the ML model. In addition, as presented in Table 2, univariate and multivariate LR analyses were conducted to further characterize the associations between each variable and OAB, providing complementary interpretability.

**Table 2.** Univariate and multivariate logistic regression analysis of factors associated with OAB^a^.

Characteristics and variables	Non-OAB (n=3781)	OAB (n=1738)	Univariate LR^b^		Multivariate LR	
			OR^c^ (95% CI)	*P* value	OR (95% CI)	*P* value
Age (y), mean (SD)	48.0 (16.3)	59.1 (15.5)	1.04 (1.04‐1.05)	<.001	1.04 (1.03‐1.05)	<.001
Race, n (%)						
Mexican American	529 (14)	233 (13.4)	—^d^	—	—	—
Other Hispanic	461 (12.2)	182 (10.5)	0.90 (0.71‐1.13)	.35	0.80 (0.63‐1.03)	.08
Non-Hispanic White	1374 (36.3)	734 (42.2)	1.21 (1.01‐1.45)	.03	1.07 (0.87‐1.31)	.55
Non-Hispanic Black	802 (21.2)	431 (24.8)	1.22 (1.01‐1.48)	.04	1.04 (0.83‐1.29)	.74
Other race	615 (16.3)	158 (9.1)	0.58 (0.46‐0.74)	<.001	0.74 (0.57‐0.96)	.02
Education, n (%)						
Below high school	762 (20.2)	416 (23.9)	—	—	—	—
High school or comparable	778 (20.6)	404 (23.2)	0.95 (0.80‐1.13)	.57	1.08 (0.89‐1.31)	.44
College or above	2241 (59.3)	918 (52.8)	0.75 (0.65‐0.86)	<.001	1.14 (0.95‐1.36)	.16
Marital status, n (%)						
Married or living with partner	2197 (58.1)	858 (49.4)	—	—	—	—
Widowed, divorced, or separated	1584 (41.9)	880 (50.6)	1.42 (1.27‐1.59)	<.001	1.08 (0.94‐1.23)	.28
PIR^e^, mean (SD)	2.5 (1.6)	2.3 (1.5)	0.93 (0.90‐0.96)	<.001	0.94 (0.90‐0.99)	.01
Smoking, n (%)						
Never	2541 (67.2)	1062 (61.1)	—	—	—	—
Former	624 (16.5)	397 (22.8)	1.52 (1.32‐1.76)	<.001	1.09 (0.93‐1.28)	.28
Current	616 (16.3)	279 (16.1)	1.08 (0.92‐1.27)	.32	1.23 (1.03‐1.47)	.02
Alcohol, n (%)						
No	3548 (93.8)	1646 (94.7)	—	—	—	—
Yes	233 (6.2)	92 (5.3)	0.85 (0.66‐1.09)	.20	—	—
Physical activity, n (%)						
Inactive	2402 (63.5)	1138 (65.5)	—	—	—	—
Moderate	893 (23.6)	389 (22.4)	0.92 (0.80‐1.06)	.23	—	—
Vigorous	90 (2.4)	46 (2.6)	1.08 (0.75‐1.55)	.68	—	—
Both moderate and vigorous	396 (10.5)	165 (9.5)	0.88 (0.72‐1.07)	.19	—	—
Age at menarche (y), mean (SD)	12.7(1.8)	12.6 (1.8)	0.96 (0.93‐0.99)	.01	0.95 (0.92‐0.99)	.006
Normal menstruation, n (%)						
No	1890 (50)	1330 (76.5)	—	—	—	—
Yes	1891 (50)	408 (23.5)	0.31 (0.27‐0.35)	<.001	0.84 (0.69‐1.02)	.08
No. of vaginal deliveries, mean (SD)	2.0 (1.7)	2.6 (1.9)	1.19 (1.16‐1.23)	<.001	1.06 (1.02‐1.10)	.003
Contraceptive, n (%)						
No	1182 (31.3)	551 (31.7)	—	—	—	—
Yes	2599 (68.7)	1187 (68.3)	0.98 (0.87‐1.11)	.74	—	—
Pregnancy diabetes, n (%)						
No	3449 (91.2)	1590 (91.5)	—	—	—	—
Borderline	26 (0.7)	16 (0.9)	1.33 (0.71‐2.50)	.37	—	—
Yes	306 (8.1)	132 (7.6)	0.94 (0.76‐1.16)	.54	—	—
Diabetes, n (%)						
No	3296 (87.2)	1298 (74.7)	—	—	—	—
Borderline	95 (2.5)	72 (4.1)	1.92 (1.41‐2.63)	<.001	1.17 (0.84‐1.64)	.36
Yes	390 (10.3)	368 (21.2)	2.40 (2.05‐2.80)	<.001	1.24 (1.01‐1.52)	.04
Glycohemoglobin (%), mean (SD)	5.7 (1.0)	6.1 (1.3)	1.32 (1.26‐1.40)	<.001	1.05 (0.98‐1.12)	.18
WBC^f^ (×10^9^/L), mean (SD)	7.2 (2.2)	7.3 (2.2)	1.02 (0.99‐1.04)	.22	—	—
Lymphocytes (×10^9^/L), mean (SD)	2.2 (1.0)	2.2 (0.8)	0.97 (0.91‐1.04)	.41	—	—
Monocytes (×10^9^/L), mean (SD)	0.5 (0.2)	0.5 (0.2)	1.64 (1.21‐2.24)	.002	0.82 (0.58‐1.17)	.28
Platelets (×10^9^/L), mean (SD)	252.2 (62.6)	248.0 (65.1)	1.00 (1.00‐1.00)	.02	1.00 (1.00‐1.00)	.34
BMI (kg/m^2^), mean (SD)	29.3 (7.6)	31.8 (8.0)	1.04 (1.03‐1.05)	<.001	1.04 (1.03‐1.05)	<.001
SBP^g^, mean (SD)	121.7 (18.5)	129.5 (19.8)	1.02 (1.02‐1.02)	<.001	1.00 (1.00‐1.01)	.39
DBP^h^, mean (SD)	69.7 (11.7)	68.9 (13.3)	0.99 (0.99‐1.00)	.01	1.00 (0.99‐1.00)	.55

a OAB: overactive bladder.

b LR: logistic regression.

c OR: odds ratio.

d Not available.

e PIR: ratio of family income to poverty threshold.

f WBC: white blood cell.

g SBP: systolic blood pressure.

h DBP: diastolic blood pressure.

### Model Explanation

**Figure 3.**
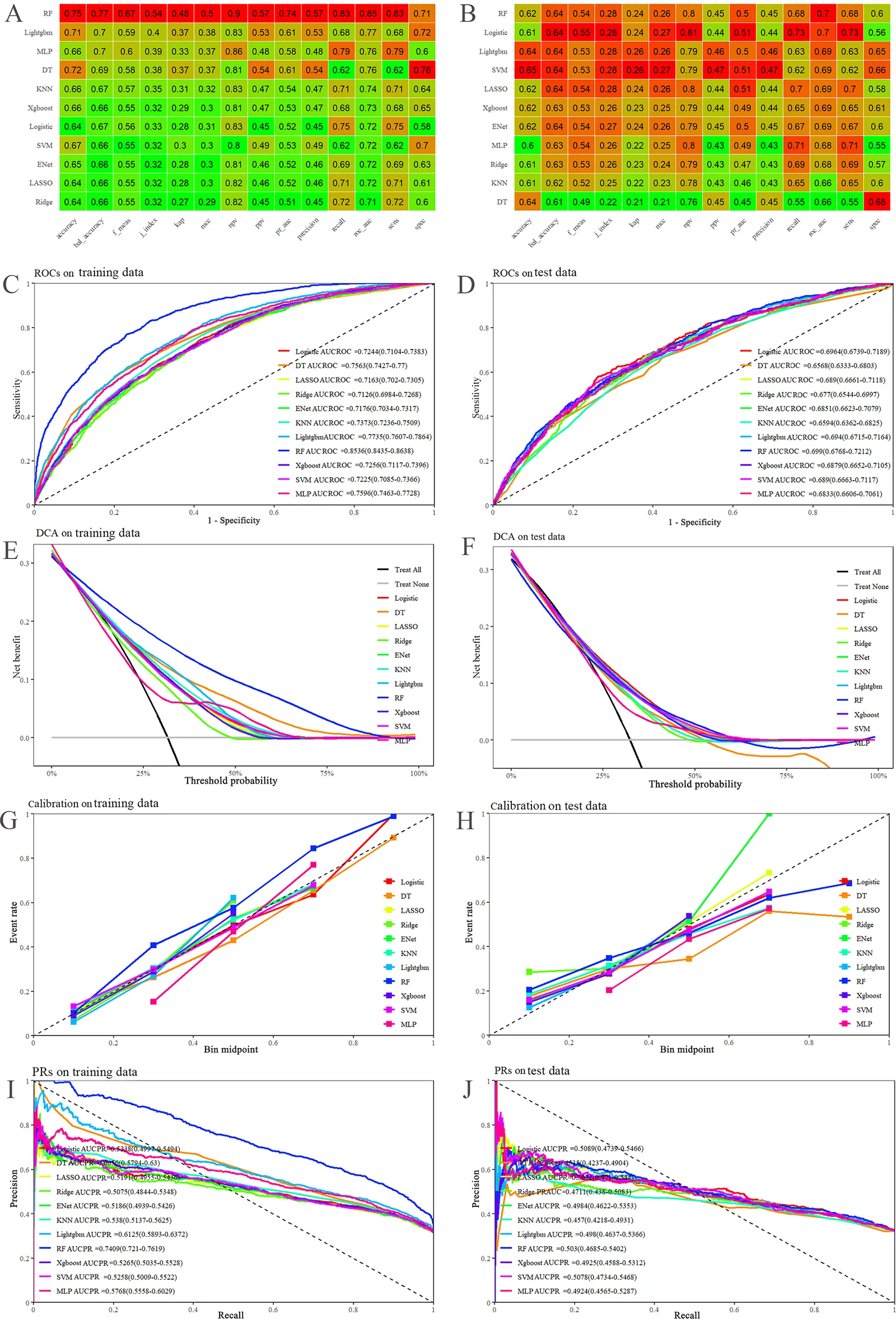
Performance evaluation, clinical use, and validation of the 11 machine learning models. The heatmap uses a green-to-red color gradient, with greener colors indicating lower values and redder colors indicating higher values. (A) Comprehensive performance metric heatmap on the training dataset; (B) comprehensive performance metric heatmap on the test dataset; (C) receiver operating characteristic (ROC) curves with area under the curve (AUROC) values on the training dataset; (D) ROC curves with AUROC values on the test dataset; (E) decision curve analysis (DCA) representing clinical net benefits across various threshold probabilities on the training dataset; (F) DCA curves on the test dataset; (G) calibration curves demonstrating the consistency between predicted risks and actual observed urological event rates on the training dataset; (H) calibration curves on the test dataset; (I) precision-recall (PR) curves with area under the PR curve (PRAUC) values on the training dataset; (J) PR curves with PRAUC values on the test dataset. DT: decision tree; ENet: elastic net; KNN: k-nearest neighbor; LASSO: least absolute shrinkage and selection operator; LightGBM, light gradient boosting machine; Logistic: logistic regression; MLP: multilayer perceptron; Ridge: ridge regression; RF: random forest; SVM: support vector machine; XGBoost: eXtreme gradient Boosting; boosting.

Five cycles of 5-fold internal cross-validation were performed to train 11 ML models. To rigorously determine the most robust predictive architecture, a comprehensive benchmark was conducted across multiple clinical validation dimensions, including discriminative power, clinical net benefit, and calibration alignment (Figure 3). As illustrated in Figures 3A and 3B, the performance metrics of all 11 algorithms were systematically compared. In terms of discriminative ability, the ROC analysis revealed that the RF model achieved the highest predictive performance, with an AUROC of 0.8536 (95% CI 0.8435‐0.8638) on the training set (Figure 3C) and 0.6999 (95% CI 0.6768‐0.7212) on the test set (Figure 3D). This superior performance of the RF model was further corroborated by the PR curves, yielding a top-tier area under the PR curve of 0.7409 on the training set (Figure 3I) and 0.5031 on the test set (Figure 3J). Concurrently, the XGBoost model demonstrated highly competitive and stable diagnostic performance, securing a test set AUROC of 0.6879 (95% CI 0.6652‐0.7105).

Beyond statistical classification accuracy, decision curve analysis was introduced to evaluate the clinical use of the developed algorithms. Across a wide range of threshold probabilities, both the RF and XGBoost models consistently demonstrated superior net benefits over the “treat-all” or “treat-none” reference strategies in both datasets, establishing their pragmatic value for clinical decision-making (Figures 3E and 3F). Furthermore, urological calibration curves were plotted to examine the concordance between the predicted risk scores and the actual observed OAB outcomes. As shown in Figure 3G and H, the calibration trajectories for the primary models (RF and XGBoost) closely adhered to the 45-degree ideal line.

**Figure 4.**
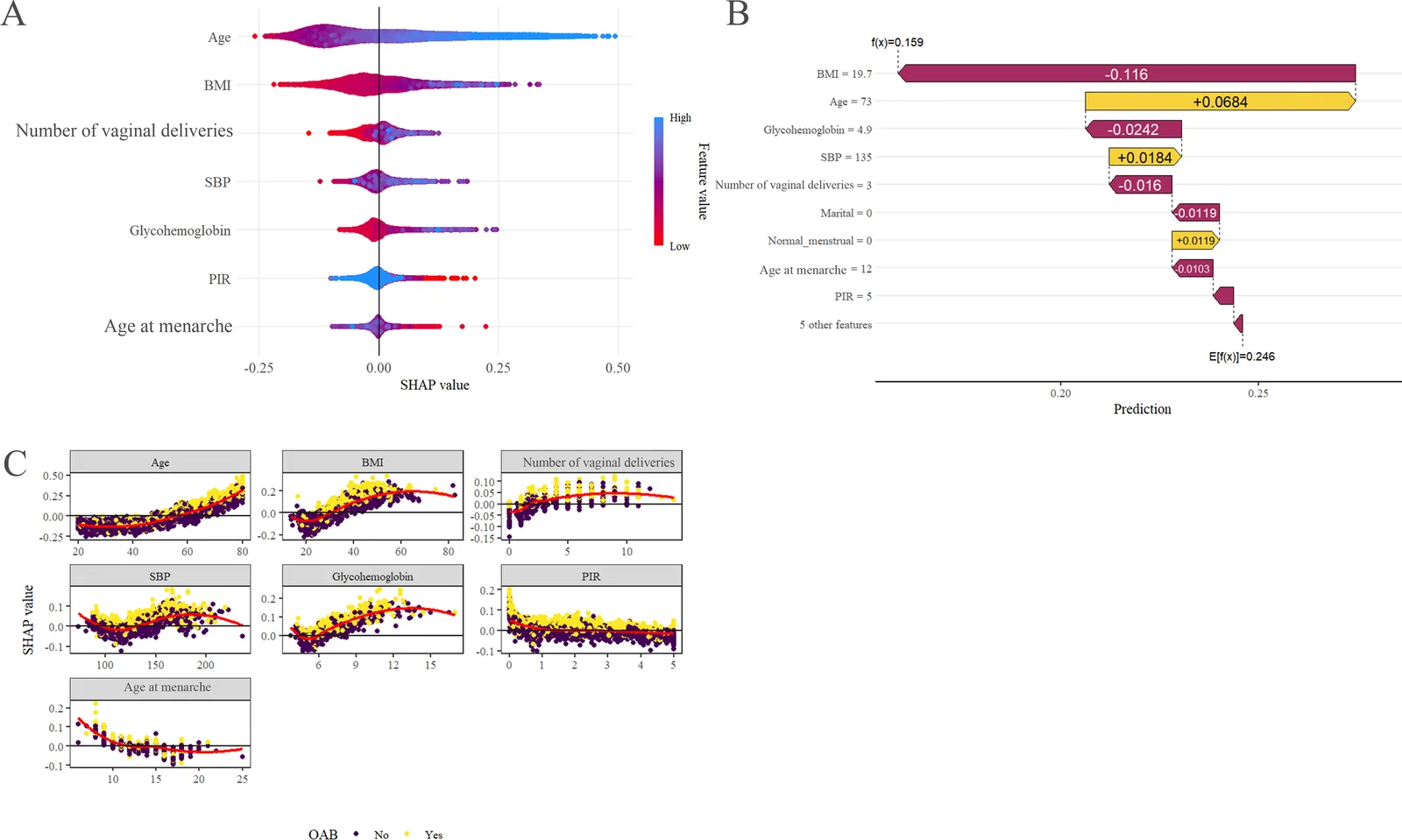
Explainable machine learning analysis using Shapley Additive Explanations (SHAP) local and global interpretations. (A) SHAP beeswarm plot demonstrating the global importance and distribution of feature contributions for the 7 continuous predictors among the 14 variables retained by least absolute shrinkage and selection operator (LASSO) regression (age, BMI, number of vaginal deliveries, systolic blood pressure [SBP], glycohemoglobin, ratio of family income to poverty threshold [PIR], and age at menarche), arranged in descending order of their mean absolute SHAP values; (B) SHAP waterfall plot illustrating the step-by-step feature attribution and quantitative contribution values for a single representative patient; (C) SHAP dependence plots revealing the continuous nonlinear relationships and distribution trends between specific variables (age, BMI, number of vaginal deliveries, SBP, glycohemoglobin, PIR, and age at menarche) and their corresponding SHAP values. OAB: overactive bladder.

The RF model’s output was interpreted using the SHAP approach, which computed each variable’s influence on the prediction. This interpretable approach provides 2 types of explanations: a local explanation at the individual level and an overall explanation of the model at the characteristic level. As depicted in the SHAP beeswarm plot (Figure 4A), the feature importance and the direction of their impact for the 7 continuous predictors among the 14 variables retained by LASSO regression were summarized in descending order of their mean absolute SHAP values: age, BMI, number of vaginal deliveries, SBP, glycohemoglobin, PIR, and age at menarche. Based on the mean absolute SHAP values, the global importance ranking of the 14 variables retained by LASSO regression was as follows: age (0.119), BMI (0.060), normal menstrual status (0.025), number of vaginal deliveries (0.024), SBP (0.022), glycohemoglobin (0.022), PIR (0.017), diabetes (0.012), age at menarche (0.012), contraceptive use (0.007), marital status (0.007), smoking (0.007), physical activity (0.005), and pregnancy diabetes (0.004). Higher values of age, BMI, SBP, and number of vaginal deliveries, along with lower values of PIR, contributed to an increased probability of OAB in the prediction model.

To closely inspect the model’s decision-making process at the individual level, a SHAP waterfall plot (Figure 4B) illustrates the quantitative contribution of each variable for a representative patient (predicted probability=0.150, baseline=0.243). In this clinical case, a BMI level of 19.7 (–0.118) kg/m^2^ contributed the most significant negative effect to the prediction, while an age of 73 (+0.0884) years, glycohemoglobin of 6.0 (+0.0242), and SBP of 115 (+0.0194) mm Hg pushed the model outcome toward higher OAB risk.

Furthermore, to capture the exact dose-response trends and the continuous distribution of features, SHAP dependence plots were constructed for key continuous variables (Figure 4C). These scatter plots demonstrate how the SHAP values vary across different ranges of each risk factor, color-coded by the actual OAB status, thereby visually uncovering the potential nonlinear interactions within the prediction model.

### Dose-Response Relationship

**Figure 5.**
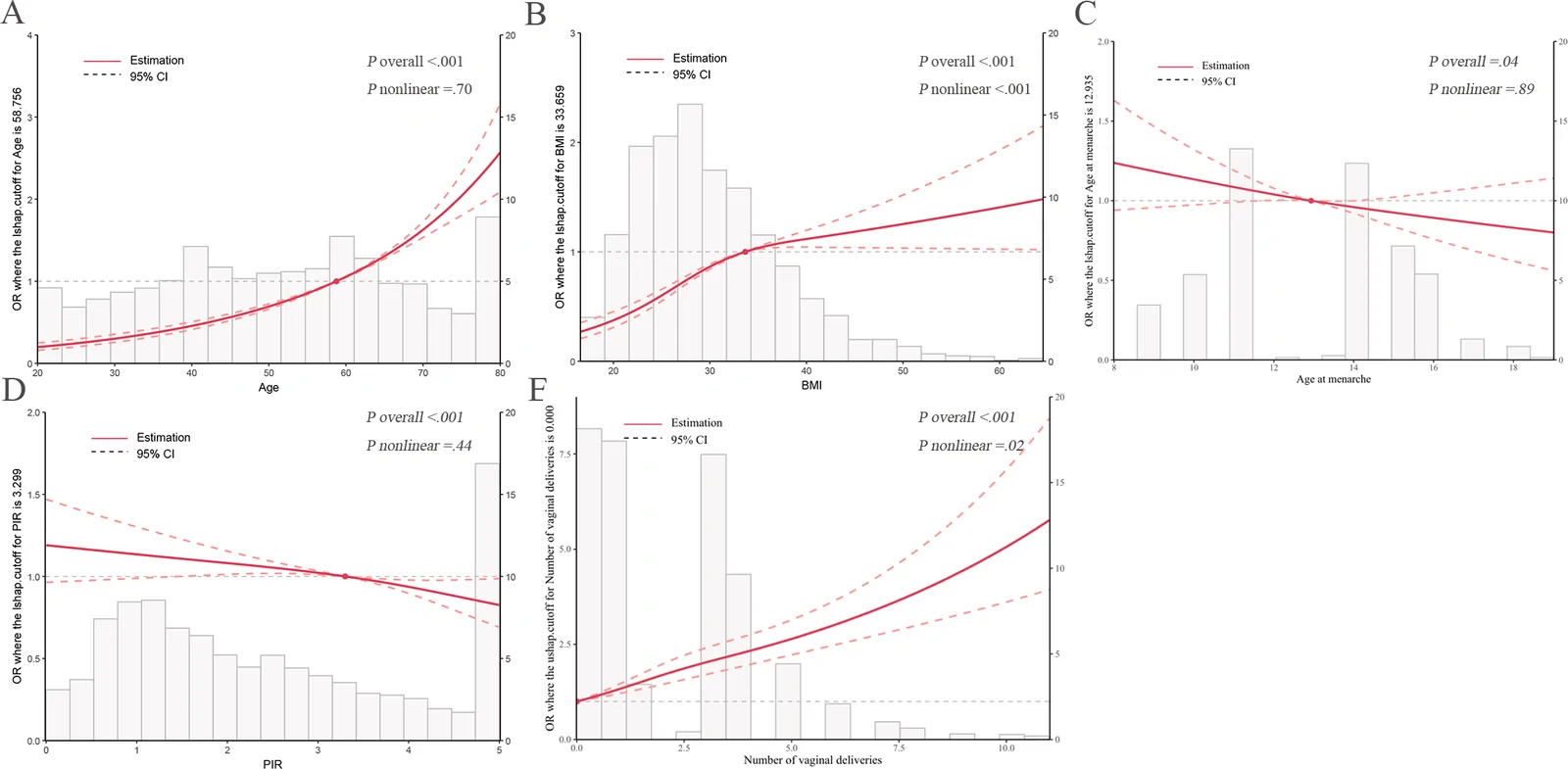
Plots using restricted cubic spline (RCS) for various continuous variables. The nonlinear correlations between each continuous variable and overactive bladder (OAB) are depicted in these figures. (A) Age, (B) BMI, (C) age at menarche, (D) ratio of family income to poverty threshold (PIR), and (E) number of vaginal deliveries are among the specific continuous variables. lshap: L-shaped association; OR: odds ratio; ushap: U-shaped association.

As shown in Figure 5, the dose-response relationship analysis was conducted on the 5 continuous predictors that remained significantly associated with OAB in multivariable logistic regression (age, BMI, PIR, age at menarche, and number of vaginal deliveries). The analysis revealed nonlinear relationships between BMI, number of vaginal deliveries, and OAB (*P*-overall <.05; *P*-nonlinear <.05). Furthermore, it was observed that when BMI values exceeded 33.659 kg/m² and the number of vaginal deliveries exceeded 0, the risk of OAB increased rapidly. Linear relationships were identified between age, age at menarche, PIR, and OAB (*P*-overall <.05; *P*-nonlinear >.05). It was observed that when the female age exceeded 58 years, the risk of developing OAB increased significantly. Additionally, it was observed that for females under the age of 13 years, earlier menstruation was associated with an elevated risk of OAB, which gradually decreased with increasing age. Furthermore, it was observed that when PIR was less than 3.299, it was identified as a risk factor for OAB, and the risk increased concomitantly with decreasing PIR.

### Interaction Analysis

**Figure 6.**
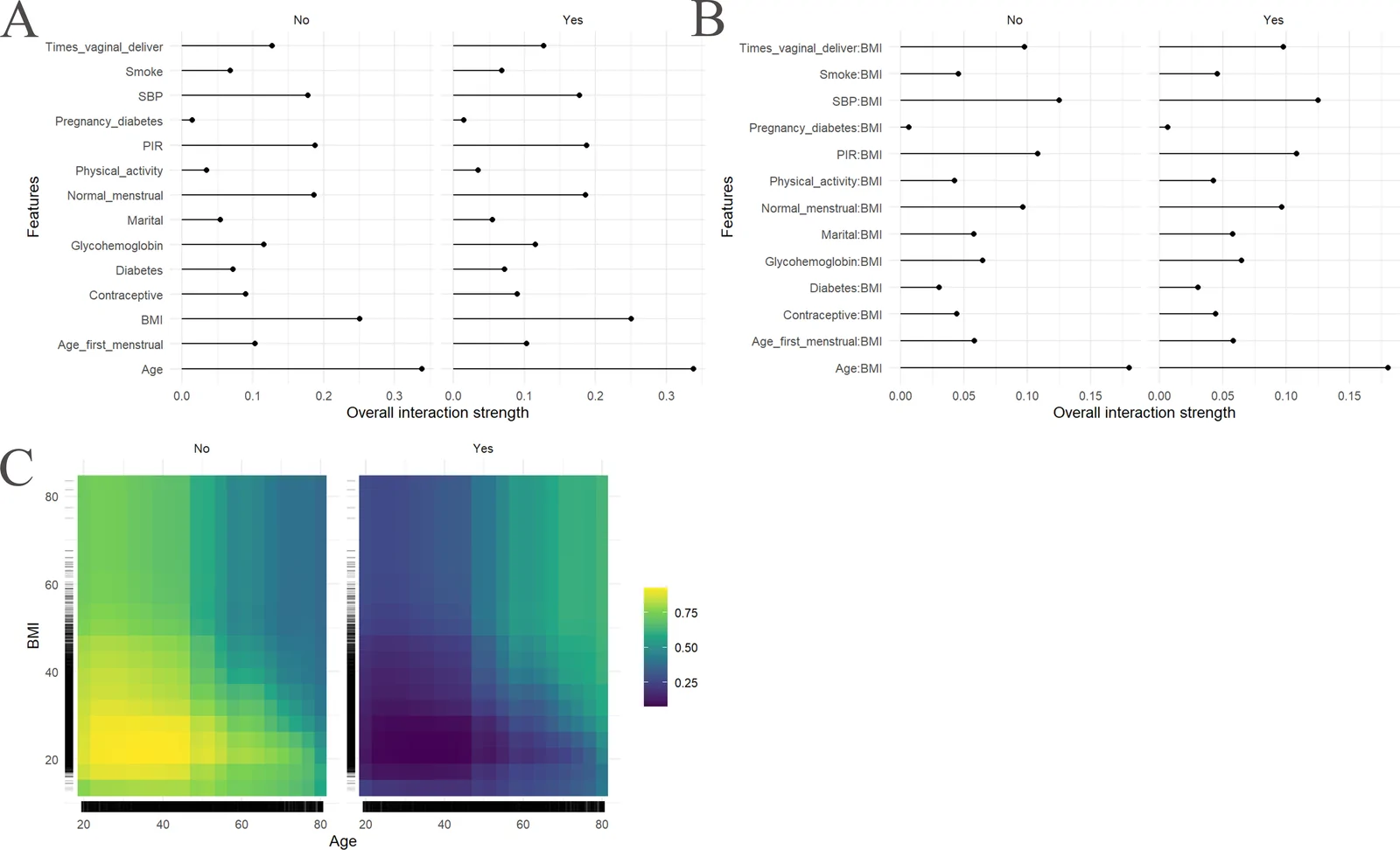
Feature interaction analysis based on Friedman *H*-statistic. (A) Overall interaction strength representing the total nonlinear interaction profile of each individual feature with all other variables, stratified by urological cohorts (No vs Yes); (B) pairwise interaction strength specifically evaluating the synergistic combinations between BMI and other clinical predictors; (C) 2-way partial dependence heatmap illustrating the joint synergistic effect of age and BMI on the predicted risk probability of overactive bladder. PIR: ratio of family income to poverty threshold; SBP: systolic blood pressure.

To further explore the potential nonlinear relationships and synergy among the predictors, a formal feature interaction analysis based on Friedman *H*-statistic was performed using the most influential variables from the final model (Figure 6). As illustrated in Figure 6A, the overall interaction strength of each feature with all other variables was quantified and stratified by urological subgroups (No vs Yes). Notably, age and BMI exhibited the highest overall interaction strengths, followed closely by PIR, number of vaginal deliveries, and normal menstrual cycle, indicating that these major risk factors do not act in isolation but possess substantial joint compounding effects on OAB manifestation.

Furthermore, since BMI was identified as a paramount driver with high connectivity, we evaluated the specific pairwise interaction strengths between BMI and all other remaining features (Figure 6B). The pairwise analysis revealed that the interaction between age and BMI (age:BMI) yielded the most pronounced synergistic effect, followed by SBP:BMI and PIR:BMI. To visually and intuitively decode this top-ranking interaction, a 2-way partial dependence plot was constructed to illustrate the joint effect of age and BMI on the predicted OAB risk matrix (Figure 6C). The heatmap demonstrates that the predicted probability of OAB peaks sharply within the combined stratum of advanced age and elevated BMI, providing strong statistical evidence of their nonlinear synergistic acceleration in OAB pathogenesis.

## Discussion

### Principal Findings

In a pioneering attempt, the potential risk factors of OAB in female participants were predicted using NHANES data combined with ML techniques. Furthermore, particular attention was paid to the influence of female reproductive factors on OAB. The findings from our comprehensive suite of univariate and multivariate LR analyses, along with LASSO, RCS, and ML methodologies, unanimously demonstrated a pronounced correlation between age, BMI, PIR, age at menarche onset, the frequency of vaginal deliveries, and the increased risk of OAB, aligning with prior research findings.

Extensive investigations have revealed that the incidence rate of OAB among females increases after the age of 40 to 50 years, corresponding with the onset of menopause [18,19]. A causal relationship has been postulated, proposing that the depletion of sex hormones during menopause may predispose women to OAB [18], a finding that aligns with our results. The findings suggest that the risk of developing OAB increases for female participants over the age of 58 years. This discrepancy with prior studies may stem from differences in study populations and sample characteristics. Obesity, a well-established critical risk factor for OAB, has garnered extensive validation. A study of 206 overweight female participants found that individuals with a body fat percentage exceeding 32% had a 95% higher risk of OAB compared to those with a body fat percentage at or below 32% [20]. A prospective study found that overweight women had a 5.8-fold increased risk of severe OAB, while obese women showed an 18.6-fold increase compared to those with a normal BMI [21]. The RCS analysis of the BMI-OAB relationship was conducted, and the results indicated that the risk of OAB increased proportionally with higher BMI. Of particular interest is the observation that, upon attaining a BMI value of 33.659 kg/m², the odds ratio transitioned from a subunity value to a value that exceeded unity. This clinical threshold of 33.659 kg/m² falls into the obesity class I category, suggesting a nonlinear cumulative effect where the bladder’s compensatory mechanisms are overwhelmed beyond this level. From a biological plausibility perspective, this sharp increase in risk may be explained by critical changes in mechanical and metabolic stresses. The occurrence of OAB may be caused by mechanical factors related to obesity, such as increased abdominal and bladder pressure [22]. Additionally, leptin secreted by visceral adipose tissue and inflammatory cytokines may lead to noradrenergic sympathetic activity and urothelial stimulation, which could be associated with the development of OAB [22,23].

Additionally, PIR serves as a proxy for the economic deprivation level of the study participants. This study found that PIR was a significant factor in precipitating OAB occurrence. When PIR was below 3.299, an inverse relationship was observed: lower PIR values corresponded to a higher likelihood of OAB, consistent with earlier findings [24,25]. This inverse relationship suggests that economic constraints may delay diagnosis and exacerbate symptoms. As reported, medical costs for individuals with OAB in the United States were more than 2.5 times higher than for those without OAB [26].

Previous studies have examined the relationship between age at menarche and urinary incontinence, but a gap remains in the existing literature regarding age at menarche and OAB. We undertook a pioneering study to investigate this relationship. The findings of this investigation revealed that earlier age of menarche onset was concomitant with an elevated risk of OAB manifestation. The risk of OAB decreased as the age at menarche exceeded 13 years. Studies have reported that early puberty onset leads to prolonged estrogen exposure, affecting hormonal sensitivity in the bladder and pelvic floor tissues, and influencing detrusor stability [27]. Furthermore, an earlier age at menarche is significantly associated with a higher risk of obesity in adulthood [28], and obesity is a known major risk factor for OAB. Vaginal delivery has been shown to increase the risk of OAB, peaking 5 years after the first delivery [29-31]. Our research findings have corroborated this observation, and the outcomes of our RCS-based analysis have indicated that the risk of OAB progressively escalates when the delivery count surpasses zero. To better understand this association, several interconnected pathophysiological mechanisms must be considered. First, vaginal childbirth can lead to significant neuromuscular injury, particularly mechanical stretching or compression of the pudendal nerve during the second stage of labor. This partial denervation of the pelvic floor muscles and detrusor can result in altered bladder sensations and subsequent detrusor overactivity due to axonal degeneration [32]. Second, connective tissue trauma plays a vital role. Mechanical distension during labor can lacerate or overstretch the pelvic organ support systems, such as the endopelvic fascia and levator ani muscle complex [33]. This collagen disruption compromises pelvic structural support, altering the anatomical position of the bladder neck and predisposing it to hypermobility, which can trigger involuntary detrusor contractions. Lastly, the prolonged mechanical compression of the fetal head against the vaginal wall can induce focal ischemia-reperfusion injury in the bladder and pelvic floor tissues [34]. The subsequent generation of reactive oxygen species and chronic inflammatory cytokines can lead to urothelium denudation and detrusor instability, further aggravating OAB symptoms.

Clinically, the primary predictors and synergies highlighted by our models underscore that OAB is a multifactorial disorder driven by interconnected mechanical (vaginal deliveries) and metabolic (BMI, glycohemoglobin) pathways. Consequently, rather than adopting a generalized approach, clinicians should leverage these risk profiles to implement phenotype-specific management, combining targeted pelvic floor rehabilitation with strict metabolic counseling to optimize preventive urological care.

This study has several strengths: first, this is the first study to predict OAB risk using the NHANES database. We used multiple methodologies (univariate or multivariate LR, RCS, and 11 ML frameworks) to analyze risk factors for OAB, focusing on female reproductive factors. Second, the data supporting this research were procured from the NHANES database, which contains exceptionally comprehensive population data, including demographic specifics and physical examination particulars. The RF model, demonstrating superior predictive performance, achieved an AUROC of 0.8536 in the training set and 0.6999 in the testing set. This model evidences moderate predictive capability. Although the RF model (testing AUROC=0.6999) and the highly competitive XGBoost model (testing AUROC=0.6879) developed in this study have not reached an excellent level (AUROC >0.8), their performance is better than the traditional LR model and shows a moderate discriminative ability. This suggests that the model can be integrated into clinical and public health practices as a tool for preliminary screening or risk stratification. For example, in community health screening or gynecological or urological outpatient clinics, a simple questionnaire (collecting age, BMI, income status, age at menarche, and delivery history) can be used to quickly calculate the OAB risk score for female participants. For individuals identified as high-risk, more detailed evaluations (such as voiding diaries or urological tests) can be recommended, thereby achieving early warning and targeted interventions to avoid symptom worsening. For women of reproductive age, this model can also be used for preconception or prenatal counseling to inform them of the potential long-term bladder health risks of multiple vaginal deliveries.

This study has several limitations that warrant consideration. First, the retrospective observational design, based on NHANES data, precludes establishing causality between identified risk factors (eg, age, BMI, age at menarche, etc) and OAB, as reverse causality—such as OAB symptoms influencing lifestyle factors like BMI—cannot be excluded, potentially leading to ambiguous interpretations of risk factor associations. Longitudinal cohort studies tracking these variables over time could clarify temporal relationships and confirm causality. Second, reliance on self-reported NHANES questionnaires introduces recall bias, particularly for variables like age at menarche, which may result in misclassification and attenuate or exaggerate associations with OAB, thus compromising result reliability. Future studies could incorporate objective measures, such as medical records or validated diagnostic tools, to enhance data accuracy. Finally, the absence of environmental (eg, occupational exposure) and genetic data limits our ability to account for potential confounders or modifiers of OAB risk, such as lifestyle factors influencing BMI or genetic predispositions affecting bladder function, potentially leading to an incomplete risk profile. While these limitations exist, they also point to valuable directions for future improvement. To enhance the model’s performance, future studies could first incorporate richer predictors, such as detailed pelvic floor electrophysiological data, bladder ultrasound parameters, genetic markers related to connective tissue metabolism, and specific quality of life or behavioral psychology data. Additionally, exploring more advanced urological algorithms and ensemble strategies, such as using deep learning to process complex feature interactions or adopting stacking techniques for model fusion, could further optimize prediction. Finally, conducting external validation in independent cohorts across different geographical and ethnic populations remains essential to verify the model’s generalizability. Integrating detailed lifestyle questionnaires and genetic profiling, such as genome-wide association studies, in future research could provide a more comprehensive understanding of OAB etiology.

### Conclusions

In this study, age, BMI, PIR, age at menarche, and the number of vaginal deliveries were identified as significant risk factors for OAB. The RF model demonstrated moderate predictive accuracy, suggesting potential utility for early screening and risk stratification of OAB in community health and primary outpatient settings; further external validation and clinical utility assessment are warranted before clinical application.

## Supplementary material

10.2196/80133 Multimedia Appendix 1 Overview of missing data, covariate correlation analysis, Overactive Bladder Symptom Score (OABSS), detailed descriptions of NHANES variables, and comparisons of participant characteristics before and after multiple imputation in the training and test datasets.

10.2196/80133 Checklist 1 TRIPOD+AI checklist.

## Abbreviations

AUROC: area under the receiver operating characteristic curve
KNN: k-nearest neighbor
LASSO: least absolute shrinkage and selection operator
LR: logistic regression
ML: machine learning
NCHS: National Center for Health Statistics
NHANES: National Health and Nutrition Examination Survey
OAB: overactive bladder
PIR: ratio of family income to poverty threshold
PR: precision-recall
RCS: restricted cubic spline
RF: random forest
SBP: systolic blood pressure
SHAP: Shapley Additive Explanations
TRIPOD+AI: Transparent Reporting of a multivariable prediction model for Individual Prognosis Or Diagnosis with Artificial Intelligence
XGBoost: eXtreme gradient boosting
