# Correction: A Machine Learning–Based Model to Predict Overactive Bladder Risk Among US Women: Evidence From the National Health and Nutrition Examination Survey 2011-2018

**DOI:** 10.2196/113546

**Published:** 2026-11-06

**Authors:** Guoqiang Huang, Shuangquan Lin

**Affiliations:** 1 Department of Urology, The Second Affiliated Hospital of Nanchang University, 1 Mingde Road, Nanchang, Jiangxi Province, 330200, China, 86 15727561896

In “A Machine Learning–Based Model to Predict Overactive Bladder Risk Among US Women: Evidence From the National Health and Nutrition Examination Survey 2011-2018” [1], the authors noted the following corrections in response to the letter to the editor, authors’ reply, and a full rereview of the published article and the analysis code conducted by the authors.

First, the Figure 2B caption has been corrected from the following:

*Ten-fold cross-validation with the minimum criterion selected the optimal λ value. The relationship between log(λ) and partial likelihood deviance (binomial deviance) is shown. Dashed vertical lines indicate the minimum mean squared error (MSE) and 1 SD from the minimum MSE (1-SE criterion), defining the optimal λ. Seven predictor variables corresponding to the log(λ) value with the minimum MSE were retained.*

The caption now reads:

*Ten-fold cross-validation with the minimum criterion selected the optimal λ value. The relationship between −log(λ) and binomial deviance is shown. The dashed vertical lines indicate the minimum binomial deviance (right line; λ=0.00550, corresponding to −log(λ)≈5.2) and 1 SE from the minimum (left line; λ=0.02933, corresponding to −log(λ)≈3.5). Variable selection was based on the minimum criterion: 14 predictor variables corresponding to the λ value with the minimum binomial deviance (λ=0.00550) were retained.*

Second, the Figure 4 caption has been clarified from the following:

*Figure 4. Explainable machine learning analysis using Shapley Additive Explanations (SHAP) local and global interpretations. (A) SHAP beeswarm plot demonstrating the global importance and distribution of feature contributions, arranged in descending order; (B) SHAP waterfall plot illustrating the step-by-step feature attribution and quantitative contribution values for a single representative patient; (C) SHAP dependence plots revealing the continuous nonlinear relationships and distribution trends between specific variables (age, BMI, number of vaginal deliveries, systolic blood pressure [SBP], glycohemoglobin, ratio of family income to poverty threshold [PIR], and age at menarche) and their corresponding SHAP values. OAB: overactive bladder.*

The caption now reads:

*Figure 4. Explainable machine learning analysis using Shapley Additive Explanations (SHAP) local and global interpretations. (A) SHAP beeswarm plot demonstrating the global importance and distribution of feature contributions for the 7 continuous predictors among the 14 variables retained by least absolute shrinkage and selection operator (LASSO) regression (age, BMI, number of vaginal deliveries, systolic blood pressure [SBP], glycohemoglobin, ratio of family income to poverty threshold [PIR], and age at menarche), arranged in descending order of their mean absolute SHAP values; (B) SHAP waterfall plot illustrating the step-by-step feature attribution and quantitative contribution values for a single representative patient; (C) SHAP dependence plots revealing the continuous nonlinear relationships and distribution trends between specific variables (age, BMI, number of vaginal deliveries, SBP, glycohemoglobin, PIR, and age at menarche) and their corresponding SHAP values. OAB: overactive bladder.*

Third, the sentence reporting the significant predictors in the Results section of the Abstract has been corrected to state the selection criterion. The sentence has been revised from the following:

*Five variables were identified as significant predictors.*

The sentence now reads:

*Five continuous predictors—age, BMI, PIR, age at menarche, and number of vaginal deliveries—remained significantly associated with OAB in multivariable logistic regression and were carried forward to the dose-response analysis.*

Fourth, the sentence reporting the top 3 Shapley Additive Explanations (SHAP) contributors in the Abstract has been corrected from the following:

SHAP analysis identified age, BMI, and the number of vaginal deliveries as the top 3 contributors to OAB risk.

The sentence now reads:

*SHAP analysis identified age, BMI, and normal menstrual status as the top 3 contributors to OAB risk.*

Fifth, the description of Figure 4A in the Results has been corrected from the following:

*As depicted in the SHAP beeswarm plot (Figure 4A), the global feature importance and the direction of their impact were summarized in descending order of their mean absolute SHAP values: age, BMI, number of vaginal deliveries, SBP, glycohemoglobin, PIR, and age at menarche.*

The sentence now reads:

*As depicted in the SHAP beeswarm plot (Figure 4A), the feature importance and the direction of their impact for the 7 continuous predictors among the 14 variables retained by LASSO regression were summarized in descending order of their mean absolute SHAP values: age, BMI, number of vaginal deliveries, SBP, glycohemoglobin, PIR, and age at menarche.*

Sixth, the Methods statement on the variables investigated in the dose-response analysis has been corrected from the following:

*The SHAP (Shapley Additive Explanations) values of the top 5 variables were investigated to ascertain the dose-response association between independent risk variables and OAB.*

The sentence now reads:

*The Shapley Additive Explanations (SHAP) values of the 5 continuous predictors that remained significantly associated with OAB in multivariable logistic regression (age, BMI, PIR, age at menarche, and number of vaginal deliveries) were investigated to ascertain the dose-response association between independent risk variables and OAB.*

Seventh, the statement in the Dose-Response Relationship section on the variables analyzed has been corrected from the following:

*As shown in Figure 5, the dose-response relationship analysis was conducted on the top 5 variables ranked with the assistance of RCS.*

The statement now reads:

*As shown in Figure 5, the dose-response relationship analysis was conducted on the 5 continuous predictors that remained significantly associated with OAB in multivariable logistic regression (age, BMI, PIR, age at menarche, and number of vaginal deliveries).*

Eighth, the last sentence of the Conclusions has been corrected from the following:

*The RF model demonstrated good predictive accuracy and practical clinical applicability, serving as a noninvasive, cost-effective tool for early screening and risk stratification in community health and primary outpatient settings.*

The sentence now reads:

*The RF model demonstrated moderate predictive accuracy, suggesting potential utility for early screening and risk stratification of OAB in community health and primary outpatient settings; further external validation and clinical utility assessment are warranted before clinical application.*

Ninth, the Conclusions section of the Abstract has been corrected from the following:

*Integrating ML with SHAP interpretability provides a robust predictive tool for OAB, facilitating early identification and clinical management.*

The Conclusions section now reads:

*Integrating ML with SHAP interpretability provides a predictive tool for OAB with moderate predictive accuracy; further external validation and clinical utility assessment are warranted before clinical application.*

Tenth, two statements in the Discussion section were overstating model performance.

The first statement was revised from the following:

*This model evidences a robust predictive capability.*

This statement now reads:

*This model evidences moderate predictive capability.*

The second statement was revised from the following:

*their performance is better than the traditional LR model and shows a moderate and robust discriminative ability.*

This statement now reads:

*their performance is better than the traditional LR model and shows a moderate discriminative ability.*

Eleventh, in the OAB Assessment section of the Methods, a statement has been amended to describe the outcome as symptom-defined overactive bladder (OAB). The following sentence was revised:

*OAB symptoms in the population were systematically evaluated using the renal disease-urology questionnaire from NHANES, and the Overactive Bladder Symptom Score was used to quantify the severity of OAB symptoms.*

This section now reads:

*OAB symptoms in the population were systematically evaluated using the renal disease-urology questionnaire from NHANES, and the Overactive Bladder Symptom Score was used to quantify the severity of OAB symptoms. In this study, OAB was defined on the basis of self-reported symptoms, with a total score of 3 or higher on the Overactive Bladder Symptom Score indicating OAB; because NHANES does not include urinalysis or urine culture data, urinary tract infection and other pathologies that can cause similar symptoms could not be excluded.*

Twelfth, the following sentence in the Methods section of the Abstract has been corrected to reflect the stated selection criterion:

*Fourteen variables were selected via LASSO regression for model construction, among which age, BMI, ratio of family income to poverty threshold (PIR), age at menarche, and number of vaginal deliveries were identified as the most significant clinical predictors.*

This sentence now reads:

*Fourteen variables were selected via LASSO regression for model construction, among which 5 continuous predictors—age, BMI, ratio of family income to poverty threshold (PIR), age at menarche, and number of vaginal deliveries—remained significantly associated with OAB in multivariable logistic regression and were used in the subsequent dose-response analysis.*

Thirteenth, a sentence reporting the SHAP-based global importance ranking of all 14 variables, with the corresponding mean absolute SHAP values, has been added to the Results section, immediately after the sentence starting “As depicted in the SHAP beeswarm plot (Figure 4A)...”

The following sentence was added:

*Based on the mean absolute SHAP values, the global importance ranking of the 14 variables retained by LASSO regression was as follows: age (0.119), BMI (0.060), normal menstrual status (0.025), number of vaginal deliveries (0.024), SBP (0.022), glycohemoglobin (0.022), PIR (0.017), diabetes (0.012), age at menarche (0.012), contraceptive use (0.007), marital status (0.007), smoking (0.007), physical activity (0.005), and pregnancy diabetes (0.004).*

Fourteenth, the classification of the BMI threshold in the Discussion section has been corrected from the following:

*This clinical threshold of 33.659 kg/m² falls into the class II obesity category, suggesting a nonlinear cumulative effect where the bladder’s compensatory mechanisms are overwhelmed beyond this level.*

The sentence now reads:

*This clinical threshold of 33.659 kg/m² falls into the obesity class I category, suggesting a nonlinear cumulative effect where the bladder’s compensatory mechanisms are overwhelmed beyond this level.*

Fifteenth, a sentence in the Discussion on health care costs has been corrected from the following:

*As reported, Medicare for individuals with OAB in the United States were more than 2.5 times higher than for those without OAB [26].*

The sentence now reads:

*As reported, medical costs for individuals with OAB in the United States were more than 2.5 times higher than for those without OAB [26].*

Sixteenth, the units for the complete blood count variables in Table 1 have been corrected from the following:

*WBC (%), median (IQR); Lymphocytes (%), median (IQR); Monocytes (%), median (IQR); Platelets (%), median (IQR)*

The units now read:

*WBC (×10^9^/L), median (IQR); Lymphocytes (×10^9^/L), median (IQR); Monocytes (×10^9^/L), median (IQR); Platelets (×10^9^/L), median (IQR)*

Seventeenth, the units for the complete blood count variables in Table 2 have been corrected from the following:

*WBC (%), mean (SD); Lymphocytes (%), mean (SD); Monocytes (%), mean (SD); Platelets (%), mean (SD)*

The units now read:

*WBC (×10^9^/L), mean (SD); Lymphocytes (×10^9^/L), mean (SD); Monocytes (×10^9^/L), mean (SD); Platelets (×10^9^/L), mean (SD)*

Eighteenth, the sentence summarizing the age difference between groups in the Results has been corrected from the following:

*patients with OAB exhibited an advanced age (61 y as opposed to 47 y in the training set and test set)*

The statement now reads:

*patients with OAB exhibited an advanced age (61 y vs 47 y in the training set and 61 y vs 48 y in the test set)*

Nineteenth, the Methods statement on laboratory variables has been amended to clarify that neutrophil count was collected and processed, and was entered into the least absolute shrinkage and selection operator (LASSO) regression as one of the 23 candidate variables, and that its coefficient was shrunk to zero at the selected λ so that it was not retained among the 14 variables selected for model construction.

The following statement was revised:

*laboratory examination outcomes (white blood cell count, lymphocyte count, monocyte count, platelet count, neutrophil count, and glycohemoglobin)*

The statement now reads:

*laboratory examination outcomes (white blood cell count, lymphocyte count, monocyte count, platelet count, neutrophil count, and glycohemoglobin). Neutrophil count was collected and processed, and was entered into the LASSO regression as one of the 23 candidate variables; its coefficient was shrunk to zero at the selected λ (0.00550), and it was not retained among the 14 variables selected for model construction.*

Twentieth, the header of the vaginal deliveries row in Table 2 has been corrected from the following:

*No of vaginal_deliveries, n ()*

The row header now reads:

*No. of vaginal deliveries, mean (SD)*

Twenty-first, a sentence in the Methods describing the SHAP values has been corrected from the following:

*These SHAP values quantify each feature’s effect on prediction accuracy.*

The sentence now reads:

*These SHAP values quantify each feature’s contribution to the model’s prediction.*

Twenty-second, the description of the correlation analysis in the Data Preprocessing section of the Methods has been corrected to reflect the analysis actually performed. The following sentences were revised:

*In addition, the correlation analysis of all continuous variables was performed using correlation heat maps. It was deemed that a significant correlation existed between variables with a correlation coefficient greater than 0.7, and those variables were deleted.*

The sentences now read:

*In addition, the correlation analysis of the continuous variables was performed using correlation heat maps (Figure S2 in Multimedia Appendix 1) to inspect multicollinearity. All 23 candidate variables were subsequently entered into the LASSO regression, and collinearity among the 14 variables retained by LASSO was checked in the data preprocessing for the machine learning models.*

Twenty-third, the number of candidate variables stated in the Figure 2A caption has been corrected, and the axis description has been aligned with the axis label. The caption was revised from the following:

*(A) LASSO coefficient curves for 22 variables. Coefficients are plotted on the y-axis, and log(λ) is shown on the x-axis.*

The caption now reads:

*(A) LASSO coefficient curves for 23 candidate variables. Coefficients are plotted on the y-axis, and −log(λ) is shown on the x-axis.*

The underlying analyses are unchanged; the corrections concern the reporting and interpretation of the results. The LASSO regression was performed on 23 candidate variables; 14 variables were retained at a λ of 0.00550 (minimum binomial deviance), and every reported metric derives from them. Neutrophil count was among the 23 candidates but was not retained.

The correction will appear in the online version of the paper on the JMIR Publications website together with the publication of this correction notice. Because this was made after submission to PubMed, PubMed Central, and other full-text repositories, the corrected article has also been resubmitted to those repositories.

## Abbreviations

LASSO: least absolute shrinkage and selection operator
OAB: overactive bladder
SHAP: Shapley Additive Explanations
