# Supplementary material for: A Machine Learning–Based Model to Predict Overactive Bladder Risk Among US Women: Evidence From the National Health and Nutrition Examination Survey 2011-2018
Source: JMIR Med Inform. 2026 Aug 12;14:e80133. doi: 10.2196/80133 (PMC13468769; doi:10.2196/80133)
Supplement: Supplementary file 2 [file medinform-v14-e80133-s001.docx]

**Multimedia Appendix 1**

**Figure S1. Variable missing value overview.**

**Figure S2. Correlation between covariates.**

**Figure S3. Overactive Bladder Symptom Score.**

**Table S1.** **The detail information of covariates in NHANES.**

**Table S2 Comparison of variables before and after multiple imputations on training set.**

**Table S3 Comparison of variables before and after multiple imputations on test set.**

**Figure S1. Variable missing value overview.** The x-axis displays the variables included in the study, while the y-axis shows the number of patients. The black lines indicate the missingness of variables for specific patients. This graph provides a visual understanding of the data completeness for each variable. Training set (A); Test set (B).

**Figure S2. Correlation between covariates.** Correlation plots showing Pearson correlation values of covariates. Training set (A); Test set (B). WBC: white blood cell; BMI: body mass index; SBP: systolic blood pressure; DBP: diastolic blood pressure.

**Figure S3. Overactive Bladder Symptom Score.**

**Table S1. The detail information of covariates in NHANES.**

| Variable in paper | | | Corresponds to variable information in NHANES | | | |
| --- | --- | --- | --- | --- | --- | --- |
|  | Variable in paper |  | Component | A description of the dataset | dataset | Corresponds to variable information in NHANES |
|  | OAB | Category, Yes,no | Questionnaire Data | Kidney Conditions - Urology | KIQ_U | KIQ044 |
|  |  |  |  | Kidney Conditions - Urology | KIQ_U | KIQ450 |
|  |  |  |  | Kidney Conditions - Urology | KIQ_U | KIQ480 |
| Covariates | age | number | Demographics Data | Demographic Variables & Sample Weights | DEMO | RIDAGEYR |
|  | body-mass index (BMI) | number | Examination Data | Body Measures | BMX | BMXBMI |
|  | sex | Category，male，female | Demographics Data | Demographic Variables & Sample Weights | DEMO | RIAGENDR |
|  | Race | Category, Hispanic and Latino Americans, non-hispanic black. Non-hispanic whites, other Hispanics, other races | Demographics Data | Demographic Variables & Sample Weights | DEMO | RIDRETH1 |
|  | Education level | Category, Below high school，High school or comparable，College or above | Demographics Data | Demographic Variables & Sample Weights | DEMO | DMDEDUC3 |
|  |  |  | Demographics Data | Demographic Variables & Sample Weights | DEMO | DMDEDUC2 |
|  | Marital status | Category, Married/Living with partner，Widowed/Divorced/Separated/ | Demographics Data | Demographic Variables & Sample Weights | DEMO | DMDMARTL |
|  | alcohol status | Category, Yes,no | DietaryData | Dietary Interview - Total Nutrient Intakes, First Day | DR1TOT | DR1TALCO |
|  |  |  | DietaryData | Dietary Interview - Total Nutrient Intakes, Second Day | DR2TOT | DR2TALCO |
|  | smoking status | Category, Yes,no | Questionnaire Data | Smoking - Cigarette Use | SMQ | SMQ020 |
|  |  |  | Questionnaire Data | Smoking - Cigarette Use | SMQ | SMQ040 |
|  | physical activity | Category,Inactive，Moderate，Vigorous | Questionnaire Data | Physical Activity - Individual Activities | PAQIAF | PADLEVEL |
|  |  |  | Questionnaire Data | Physical Activity | PAQ | PAQ620 |
|  |  |  | Questionnaire Data | Physical Activity | PAQ | PAQ605 |
|  | comorbid conditions | Category, Yes,no |  |  |  |  |
|  | diabetes |  | Questionnaire Data | Diabetes | DIQ | DIQ010 |
|  |  |  | Questionnaire Data | Diabetes | DIQ | DIQ050 |
|  |  |  | Questionnaire Data | Diabetes | DIQ | DID070 |
|  |  |  | Questionnaire Data | Diabetes | DIQ | DIQ070 |
|  |  |  | Laboratory Data | Glycohemoglobin | GHB | LBXGH |
|  |  |  | Laboratory Data | Plasma Fasting Glucose & Insulin | GLU | LBXGLU |
|  |  |  | Questionnaire Data | Kidney Conditions - Urology | KIQ_U | KIQ022 |
|  | women reproductive health | number | Questionnaire Data | Age_first_menstrual | RHQ | RHQ010 |
|  |  | Category, Yes,no | Questionnaire Data | Normal_menstrual | RHQ | RHQ031 |
|  |  | number | Questionnaire Data | Times_vaginal_deliver | RHQ | RHQ166 |
|  |  | Category, Yes,no | Questionnaire Data | Contraceptive | RHQ | RHQ420 |
|  |  | Category, Yes,no | Questionnaire Data | Pregnancy_diabetes | RHQ | RHQ162 |

**Table S2** Comparison of characteristics before and after multiple imputations on training set.

|  | **Before multiple imputations** | **After multiple imputations** | **p.overall** |
| --- | --- | --- | --- |
|  | ***N=5519*** | ***N=5519*** |  |
| Age | 52.0 [38.0;64.5] | 52.0 [38.0;64.5] | 1.000 |
| Race: |  |  | 1.000 |
| 0 | 762 (13.8%) | 762 (13.8%) |  |
| 1 | 643 (11.7%) | 643 (11.7%) |  |
| 2 | 2108 (38.2%) | 2108 (38.2%) |  |
| 3 | 1233 (22.3%) | 1233 (22.3%) |  |
| 4 | 773 (14.0%) | 773 (14.0%) |  |
| Education: |  |  | 1.000 |
| 0 | 1178 (21.3%) | 1178 (21.3%) |  |
| 1 | 1182 (21.4%) | 1182 (21.4%) |  |
| 2 | 3158 (57.2%) | 3159 (57.2%) |  |
| Marital: |  |  | 1.000 |
| 0 | 3054 (55.3%) | 3055 (55.4%) |  |
| 1 | 2464 (44.7%) | 2464 (44.6%) |  |
| PIR | 2.02 [1.07;3.92] | 2.12 [1.13;3.77] | 0.268 |
| Smoke: |  |  | 1.000 |
| 0 | 3599 (65.3%) | 3603 (65.3%) |  |
| 1 | 1021 (18.5%) | 1021 (18.5%) |  |
| 2 | 895 (16.2%) | 895 (16.2%) |  |
| Alcohol: |  |  | 0.521 |
| 0 | 4915 (93.8%) | 5194 (94.1%) |  |
| 1 | 325 (6.20%) | 325 (5.89%) |  |
| Physical_activity: |  |  | 1.000 |
| 0 | 3540 (64.1%) | 3540 (64.1%) |  |
| 1 | 1282 (23.2%) | 1282 (23.2%) |  |
| 2 | 136 (2.46%) | 136 (2.46%) |  |
| 3 | 561 (10.2%) | 561 (10.2%) |  |
| Age_first_menstrual | 13.0 [12.0;14.0] | 13.0 [12.0;14.0] | 0.880 |
| Normal_menstrual: |  |  | 1.000 |
| 0 | 3220 (58.3%) | 3220 (58.3%) |  |
| 1 | 2299 (41.7%) | 2299 (41.7%) |  |
| Times_vaginal_deliver | 2.00 [1.00;3.00] | 2.00 [1.00;3.00] | 0.246 |
| Contraceptive: |  |  | 0.997 |
| 0 | 1732 (31.4%) | 1733 (31.4%) |  |
| 1 | 3780 (68.6%) | 3786 (68.6%) |  |
| Pregnancy_diabetes: |  |  | 0.488 |
| 0 | 4638 (90.6%) | 5039 (91.3%) |  |
| 1 | 41 (0.80%) | 42 (0.76%) |  |
| 2 | 438 (8.56%) | 438 (7.94%) |  |
| Diabetes: |  |  | 1.000 |
| 0 | 4592 (83.2%) | 4594 (83.2%) |  |
| 1 | 167 (3.03%) | 167 (3.03%) |  |
| 2 | 758 (13.7%) | 758 (13.7%) |  |
| Glycohemoglobin | 5.60 [5.30;5.90] | 5.60 [5.30;5.90] | 0.953 |
| WBC | 6.90 [5.70;8.40] | 6.90 [5.70;8.40] | 1.000 |
| Lymphocytes | 2.10 [1.70;2.60] | 2.10 [1.70;2.60] | 1.000 |
| Monocytes | 0.50 [0.40;0.60] | 0.50 [0.40;0.60] | 1.000 |
| Platelets | 245 [207;289] | 245 [207;289] | 1.000 |
| Neutrophils | 4.00 [3.10;5.10] | 4.00 [3.10;5.10] | 1.000 |
| BMI | 28.7 [24.4;34.3] | 28.7 [24.4;34.3] | 0.977 |
| SBP | 121 [110;135] | 121 [110;135] | 0.630 |
| DBP | 70.0 [63.0;77.0] | 70.0 [63.0;77.0] | 0.954 |
| OAB | 0.00 [0.00;1.00] | 0.00 [0.00;1.00] | 1.000 |

Continuous normally distributed variables were reported as the mean ± standard deviation (SD), which were then compared via t-tests. Non-normal variables were reported as the median [interquartile range] (IQR), which were then compared via Mann-Whitney U or Kruskal-Wallis tests. Categorical variables were reported as counts (%), which were then compared via chi-square tests.

WBC: white blood cell; BMI: body mass index; SBP: systolic blood pressure; DBP: diastolic blood pressure; PIR: Ratio of family income to poverty threshold.

**Table S3** Comparison of characteristics before and after multiple imputations on test set.

|  | **Before multiple imputations** | **After multiple imputations** | **p.overall** |
| --- | --- | --- | --- |
|  | ***N=2365*** | ***N=2365*** |  |
| Age | 52.0 [39.0;65.0] | 52.0 [39.0;65.0] | 1.000 |
| Race: |  |  | 1.000 |
| 0 | 343 (14.5%) | 343 (14.5%) |  |
| 1 | 257 (10.9%) | 257 (10.9%) |  |
| 2 | 902 (38.1%) | 902 (38.1%) |  |
| 3 | 556 (23.5%) | 556 (23.5%) |  |
| 4 | 307 (13.0%) | 307 (13.0%) |  |
| Education: |  |  | 1.000 |
| 0 | 512 (21.6%) | 512 (21.6%) |  |
| 1 | 532 (22.5%) | 532 (22.5%) |  |
| 2 | 1321 (55.9%) | 1321 (55.9%) |  |
| Marital: |  |  | 1.000 |
| 0 | 1299 (54.9%) | 1299 (54.9%) |  |
| 1 | 1066 (45.1%) | 1066 (45.1%) |  |
| PIR | 1.87 [1.01;3.70] | 1.98 [1.06;3.52] | 0.374 |
| Smoke: |  |  | 1.000 |
| 0 | 1503 (63.6%) | 1503 (63.6%) |  |
| 1 | 469 (19.8%) | 469 (19.8%) |  |
| 2 | 393 (16.6%) | 393 (16.6%) |  |
| Alcohol: |  |  | 0.663 |
| 0 | 2085 (93.1%) | 2210 (93.4%) |  |
| 1 | 155 (6.92%) | 155 (6.55%) |  |
| Physical_activity: |  |  | 1.000 |
| 0 | 1550 (65.5%) | 1550 (65.5%) |  |
| 1 | 483 (20.4%) | 483 (20.4%) |  |
| 2 | 63 (2.66%) | 63 (2.66%) |  |
| 3 | 269 (11.4%) | 269 (11.4%) |  |
| Age_first_menstrual | 13.0 [12.0;14.0] | 13.0 [12.0;14.0] | 0.985 |
| Normal_menstrual: |  |  | 1.000 |
| 0 | 1387 (58.6%) | 1387 (58.6%) |  |
| 1 | 978 (41.4%) | 978 (41.4%) |  |
| Times_vaginal_deliver | 2.00 [1.00;3.00] | 2.00 [1.00;3.00] | 0.523 |
| Contraceptive: |  |  | 1.000 |
| 0 | 773 (32.7%) | 773 (32.7%) |  |
| 1 | 1592 (67.3%) | 1592 (67.3%) |  |
| Pregnancy_diabetes: |  |  | 0.725 |
| 0 | 1987 (91.1%) | 2169 (91.7%) |  |
| 1 | 13 (0.60%) | 14 (0.59%) |  |
| 2 | 182 (8.34%) | 182 (7.70%) |  |
| Diabetes: |  |  | 1.000 |
| 0 | 1956 (82.7%) | 1957 (82.7%) |  |
| 1 | 65 (2.75%) | 65 (2.75%) |  |
| 2 | 343 (14.5%) | 343 (14.5%) |  |
| Glycohemoglobin | 5.60 [5.30;6.00] | 5.60 [5.30;6.00] | 0.934 |
| WBC | 6.90 [5.60;8.40] | 6.90 [5.60;8.40] | 1.000 |
| Lymphocytes | 2.10 [1.70;2.60] | 2.10 [1.70;2.60] | 1.000 |
| Monocytes | 0.50 [0.40;0.60] | 0.50 [0.40;0.60] | 1.000 |
| Platelets | 245 [211;288] | 245 [211;288] | 1.000 |
| Neutrophils | 4.00 [3.10;5.10] | 4.00 [3.10;5.10] | 1.000 |
| BMI | 29.1 [24.6;34.4] | 29.2 [24.6;34.3] | 0.930 |
| SBP | 122 [110;135] | 122 [110;135] | 0.734 |
| DBP | 70.0 [63.0;77.0] | 70.0 [63.0;77.0] | 0.976 |
| OAB | 0.00 [0.00;1.00] | 0.00 [0.00;1.00] | 1.000 |

Continuous normally distributed variables were reported as the mean ± standard deviation (SD), which were then compared via t-tests. Non-normal variables were reported as the median [interquartile range] (IQR), which were then compared via Mann-Whitney U or Kruskal-Wallis tests. Categorical variables were reported as counts (%), which were then compared via chi-square tests.

WBC: white blood cell; BMI: body mass index; SBP: systolic blood pressure; DBP: diastolic blood pressure; PIR: Ratio of family income to poverty threshold.
